# Phylogenomic resolution of order- and family-level monocot relationships using 602 single-copy nuclear genes and 1375 BUSCO genes

**DOI:** 10.3389/fpls.2022.876779

**Published:** 2022-11-22

**Authors:** Prakash Raj Timilsena, Eric K. Wafula, Craig F. Barrett, Saravanaraj Ayyampalayam, Joel R. McNeal, Jeremy D. Rentsch, Michael R. McKain, Karolina Heyduk, Alex Harkess, Matthieu Villegente, John G. Conran, Nicola Illing, Bruno Fogliani, Cécile Ané, J. Chris Pires, Jerrold I. Davis, Wendy B. Zomlefer, Dennis W. Stevenson, Sean W. Graham, Thomas J. Givnish, James Leebens-Mack, Claude W. dePamphilis

**Affiliations:** ^1^ Department of Biology and Huck Institutes of the Life Sciences, Pennsylvania State University, University Park, PA, United States; ^2^ Department of Biology, West Virginia University, Morgantown, WV, United States; ^3^ Georgia Advanced Computing Resource Center, University of Georgia, Athens, GA, United States; ^4^ Department of Plant Biology, University of Georgia, Athens, GA, United States; ^5^ Department of Ecology, Evolution, and Organismal Biology, Biology Kennesaw State University, Kennesaw, GA, United States; ^6^ Department of Biology, Francis Marion University, Florence, SC, United States; ^7^ Department of Biological Sciences, University of Alabama, Tuscaloosa, AL, United States; ^8^ School of Life Sciences, University of Hawai’i at Mānoa, Honolulu, HI, United States; ^9^ HudsonAlpha Institute for Biotechnology, Huntsville, AL, United States; ^10^ Institut des Sciences Exactes et Appliquees (ISEA), University of New Caledonia, Noumea, New Caledonia; ^11^ Australian Centre for Evolutionary Biology and Biodiversity & Sprigg Geobiology Centre, School of Biological Sciences, University of Adelaide, Adelaide, SA, Australia; ^12^ Department of Molecular and Cell Biology, University of Cape Town, Cape Town, South Africa; ^13^ Department of Botany, University of Wisconsin-Madison, Madison, WI, United States; ^14^ Department of Statistics, University of Wisconsin–Madison, Madison, WI, United States; ^15^ Division of Biological Sciences and Bond Life Sciences Center, University of Missouri, Columbia, MO, United States; ^16^ School of Integrative Plant Sciences and L.H. Bailey Hortorium, Cornell University, Ithaca, NY, United States; ^17^ Department of Botany, University of British Columbia, Vancouver, BC, Canada; ^18^ New York Botanical Garden, New York, NY, United States

**Keywords:** phylogenomics, phylotranscriptomics, monocots, conserved single-copy genes, BUSCO, concordance analysis

## Abstract

We assess relationships among 192 species in all 12 monocot orders and 72 of 77 families, using 602 conserved single-copy (CSC) genes and 1375 benchmarking single-copy ortholog (BUSCO) genes extracted from genomic and transcriptomic datasets. Phylogenomic inferences based on these data, using both coalescent-based and supermatrix analyses, are largely congruent with the most comprehensive plastome-based analysis, and nuclear-gene phylogenomic analyses with less comprehensive taxon sampling. The strongest discordance between the plastome and nuclear gene analyses is the monophyly of a clade comprising Asparagales and Liliales in our nuclear gene analyses, versus the placement of Asparagales and Liliales as successive sister clades to the commelinids in the plastome tree. Within orders, around six of 72 families shifted positions relative to the recent plastome analysis, but four of these involve poorly supported inferred relationships in the plastome-based tree. In Poales, the nuclear data place a clade comprising Ecdeiocoleaceae+Joinvilleaceae as sister to the grasses (Poaceae); Typhaceae, (rather than Bromeliaceae) are resolved as sister to all other Poales. In Commelinales, nuclear data place Philydraceae sister to all other families rather than to a clade comprising Haemodoraceae+Pontederiaceae as seen in the plastome tree. In Liliales, nuclear data place Liliaceae sister to Smilacaceae, and Melanthiaceae are placed sister to all other Liliales except Campynemataceae. Finally, in Alismatales, nuclear data strongly place Tofieldiaceae, rather than Araceae, as sister to all the other families, providing an alternative resolution of what has been the most problematic node to resolve using plastid data, outside of those involving achlorophyllous mycoheterotrophs. As seen in numerous prior studies, the placement of orders Acorales and Alismatales as successive sister lineages to all other extant monocots. Only 21.2% of BUSCO genes were demonstrably single-copy, yet phylogenomic inferences based on BUSCO and CSC genes did not differ, and overall functional annotations of the two sets were very similar. Our analyses also reveal significant gene tree-species tree discordance despite high support values, as expected given incomplete lineage sorting (ILS) related to rapid diversification. Our study advances understanding of monocot relationships and the robustness of phylogenetic inferences based on large numbers of nuclear single-copy genes that can be obtained from transcriptomes and genomes.

## Introduction

The monocots are a large monophyletic group of angiosperms, comprising 12 orders, 77 families and about 60,000–85,000 species (Bremer et al., 2009; Chase and Reveal, 2009; Lughadha et al., 2016; Givnish et al., 2018). They underpin some of the most productive ecosystems, including grasslands (e.g., prairies and steppes) and many aquatic habitats (e.g., seagrass meadows) (Waycott et al., 2009). Human civilization depends on cereal crops such as rice, oats and wheat (e.g., Mabberley, 2008). In addition to cereals and grains, major berry crops (e.g., plantain/banana), forage/fodder species (grasses), and various stem and “root” crops (e.g., sugar cane, onion, yam tubers), collectively provide core food sources for billions of humans. Some individual species have been put to versatile uses: the coconut (*Cocos nucifera*), for example, has fruits, stems, and leaves that are important sources of food, beverages, timber, and fiber. Other monocot crops provide a rich variety of spices (e.g., vanilla, cardamom), herbs (e.g., lemongrass), and beverages (e.g., plant-based milks, beer, and many grain- and sugar-based spirits). In addition, monocots provide biofuel, feedstock (e.g., palm oil, maize, sugarcane, switchgrass), timber (bamboo, *Pandanus*), and other material for housing, thatching and lawns (multiple grass species). Monocots are also important sources of pharmaceuticals and essential oils, and they provide many attractive ornamental species, including large numbers of bulbous and cormous herbs—such as crocuses, irises, lilies, onions, and trilliums—as well as the extraordinarily diverse orchids. Some monocots are also used in culturally important ceremonies (e.g., sweetgrass use by North American indigenous peoples). Thus, monocots are arguably the most economically and socially important group of green plants (Viridiplantae).

Monocots are estimated to have originated 136–140 million years ago (Mya) (Magallon et al., 2015; Smith and Brown, 2018) and comprise about one-fourth of angiosperm species. Over the intervening time, they have evolved great diversity in ecology and growth form, including: tiny free-floating duckweeds; seagrasses; grassy, often fire-resistant herbs with parallel leaf venation; broad-leaved and gigantic herbs with net venation; resurrection plants; shrubs; vines; tall, highly lignified tree-like plants without true secondary vascular growth; tropical epiphytes; non-green mycoheterotrophs that parasitize fungi and often lurk in dense shade; and at least five species of carnivorous plants (Dahlgren et al., 1985; Kress, 1990; Givnish et al., 2005; Kress and Specht, 2005; Givnish et al., 2010; Merckx et al., 2010; Merckx et al., 2013; Givnish et al., 2016; Lam et al., 2016; Givnish et al., 2018; Lin et al., 2021). Plants within a single order may show enormous morphological diversity, as illustrated by Asparagales, Liliales, and Pandanales (The Angiosperm Phylogeny Group et al., (APG) IV, 2016; Dahlgren et al., 1985; Kubitzki et al., 1998). Monocots also exhibit substantial diversity in the size and shape of their reproductive organs, including species with the smallest flowers (*Wolffia*), the most massive unbranched (*Amorphophallus*) and branched (*Corypha*) inflorescences, and the smallest, dustlike seeds (Orchidaceae, some less than one-millionth of a gram), and the most massive seeds (*Lodoicea*, at 18 kg). Confident resolution of monocot relationships based on multiple robust lines of evidence is a critical goal of evolutionary systematics, and is essential for understanding patterns of morphological, ecological, and geographical diversification (Givnish et al., 2018).

## Phylogeny of monocots

Recent work on relationships among monocot orders and families has been based largely on DNA sequences of plastid-encoded genes or genes extracted from whole plastid genomes (plastomes) (Graham et al., 2006; Givnish et al., 2010; Soltis et al., 2011; Steele et al., 2012; Davis et al., 2014; Govindarajulu et al., 2015; Barrett et al., 2016a; Givnish et al., 2016; Lam et al., 2018; Givnish et al., 2018). Even with whole plastome sequences, some key uncertainties regarding familial and ordinal relationships, and some plastome-based inferences conflict with those based on phylogenomics analyses of nuclear gene sequences (Sass and Specht, 2010; Zeng et al., 2014; McKain et al., 2016; Sass et al., 2016; One Thousand Plant Transcriptomes (OTPT) Initiative, 2019; Baker et al., 2022). Plastome genes are inherited as a single locus (Doyle, 2022) and plastome tree-species tree discordance may be a consequence of incomplete lineage sorting, hybridization/introgression, or misspecification of substitution models (e.g., Linder and Rieseberg, 2004; Willyard et al., 2009; Sessa et al., 2012; Davis et al., 2014; Garcia et al., 2014; Davis and Xi, 2015; Vargas et al., 2017).

In this study, we use nuclear gene sequences to resolve phylogenetic relationships among all orders and almost all families of monocots. We identify 602 nuclear genes that are conserved in single-copy form across a 12-genome dataset including nine monocots and three non-monocot outgroups. We also assessed the robustness of some inferences based on the 602 conserved single-copy (CSC) genes by comparing species trees estimated using the CSC gene set and the 1,375 Benchmarking Universal Single Copy Ortholog (BUSCO) gene set (Simão et al., 2015; Waterhouse et al., 2017). BUSCO genes are typically used for genome and transcriptome quality assessments, and increasingly extracted from genome and transcriptome data for phylogenomic analyses in plants (Simão et al., 2015; Waterhouse et al., 2018; Manni et al., 2021; Zhao et al., 2021). Lastly, we use concordance factor analysis to more deeply explore branches that have been contentious in previous studies or that disagree with relationships based on analyses of genes extracted from complete plastomes.

## Materials and methods

### Taxon sampling, data collection, and sequencing

Our sampling included representatives of 72 of 77 recognized families of monocots (APG IV, 2016; the unsampled families are Blandfordiaceae, Corsiaceae, Juncaginaceae, Ripogonaceae, and Ruppiaceae); we analyzed 173 transcriptomes and 25 genomes, for a total of 198 taxa (**Table S1**). These data include 79 newly sequenced transcriptomes derived from RNA extracted from flash-frozen young leaf material (NCBI BioProjects PRJNA313089, PRJNA752894, SRP009920, PRJNA412930, and PRJNA752837). RNA was extracted following the methods described by Johnson et al. (2012). Illumina Tru-Seq libraries were constructed following the manufacturer’s protocols (Illumina, San Diego, CA, USA) and sequenced on Illumina HiSeq or NextSeq 500 platforms (**Table S1**). Additional transcriptomes and genomes were also obtained from Phytozome (Goodstein et al., 2012), Ensembl Plants (Bolser et al., 2017), NCBI (Agarwala et al., 2017), the One Thousand Plant Transcriptomes Project (1KP) (OTPT Initiative, 2019) and other genome project databases (**Tables S1-S3**).

### Transcript assembly

Quality assessments of reads and adapter contamination analysis were performed using FastQC (Andrews, 2010) and any adapters were removed with Cutadapt (Martin, 2011). The reads were trimmed from the ends at positions with three consecutive bases with scores less than Q20. After trimming, reads with median quality scores less than Q22 and more than 3 uncalled bases were removed. Any read less than 40 bp in length after filtering was also removed. Cleaned reads were assembled using the Trinity v. 2013-02-25-*de novo* assembler (Haas et al., 2013). They were then aligned back to the Trinity assembly multifasta file using Bowtie (v. 0.12.8) (Langmead, 2010). RSEM v. 1.1.21 (Li and Dewey, 2011) was used to quantify the abundance of different isoforms. The assembly was then filtered to remove isoforms that had less than 1% of FPKM (Fragments Per Kilobase of transcript per Million mapped reads). Assembled transcript sequences for each species were translated using ESTScan v. 2.1 (Iseli et al., 1999), using *Oryza sativa* gene models as the training set.

### Gene-family circumscription and assignment of transcript assemblies to orthogroups

We created a PlantTribes database (Wall et al., 2008) from protein-coding sequences extracted from the annotations to enable the global identification of conserved single copy (CSC) genes across a diverse set of monocot genomes. All protein-coding gene models from nine and three published monocot and non-monocot angiosperm genomes, respectively (**Table S2**), were clustered using OrthoMCL (Li et al., 2003) to circumscribe orthogroups approximating gene families. OrthoMCL was run with a 1E-5 BLASTP e-value cutoff and an inflation factor of 1.2. The resulting gene family scaffold comprised 24,873 orthogroups of which 602 stringently defined single copy gene families. The 602 CSC orthogroups, with exactly one gene from each of the 12 reference genomes, were used for phylogenomic analyses.

Gene sequences from transcriptome assemblies and additional genomes were assigned to orthogroups using a combination of protein BLAST and Hidden Markov Models (HMMs) using a two-step approach. Transcript assemblies were translated using ESTScan to obtain the corresponding open reading frames (ORFs) and protein translations (Iseli et al., 1999). Hmmscan v. 3.3.2 within the HMMER package (Eddy, 2011) was then used to interrogate translated sequences for each sample with orthogroup HMM profiles. Queries of the 12-genome scaffold protein database were then conducted using BLASTp v. 2.2.26 (Altschul et al., 1990) with a threshold of 1e-5. Orthogroup assignment was based on the hmmscan results, which typically corresponded to the orthogroup that included the best BLAST hit.

Transcript assemblies and genome models assigned to the 602 putatively CSC orthogroups were inspected further. Following methods used by the One Thousand Plant Transcriptome Initiative (Matasci et al. 2014; Wickett et al., 2014; OTPT Initiative, 2019), and implemented through the AssemblyPostProcessor steps in the PlantTribes toolkit (https://github.com/dePamphilis/PlantTribes), if multiple transcript assemblies from a single sample were assigned to a CSC orthogroup, they were scaffolded using the banana genome (*Musa*) as a reference. If the transcript sequence overlapped with a sequence similarity of 95% or better, a consensus sequence was retained for downstream analyses. If divergence among multiple transcript assemblies for a sample sorted to a CSC orthogroup was greater than 5%, the sequences for that sample were treated as missing data for downstream analyses of that CSC orthogroup. This scaffolding process could combine splice variants into consensus sequences or treat splice variants as paralogs when they do not align well. Similarly, for the genomes included beyond the 12 used for orthogroup construction (**Table S1**, **S3**), paralogous gene models sorted to a CSC orthogroup were also discarded. All retained transcript assemblies and scaffolds were included in multiple sequence alignments and phylogenetic analyses.

DNA and protein sequences from all taxa were brought together to create fasta files for each CSC orthogroup. Protein sequences were aligned using MAFFT v. 7.4 (Katoh and Standley, 2013), trimmed using trimAl (Capella-Gutierrez et al., 2009), and then DNA sequences were forced onto the protein alignments, all using the PlantTribes GeneFamilyAligner tool (Wafula, 2019; https://github.com/dePamphilis/PlantTribes). A maximum of 10 alignment iterations was run; for each iteration, sites in the alignments with less than 90% occupancy or sequences with gene length less than 90% of the alignment were removed, and the remaining sequences were realigned.

Species relationships were estimated using the coalescence-based gene tree summary method implemented in ASTRAL III (Zhang et al., 2018) with default settings. Input gene trees were estimated for each of the 602 CSC orthogroup alignments using RAxML v. 8.2, with analyses partitioned by codon position as below, and a GTRGAMMA model of rate variation, with 100 rapid bootstrap replicates. TreeShrink (Mai and Mirarab, 2018) on the “per-species basis” was used to identify and filter out “rogue” taxa (that is, single genes were removed from individual taxa) that exhibited significantly greater than expected variation in placement among gene trees, possibly due to sequence error resulting in out of frame indels and mistranslation, unspliced introns, contamination, or issues with paralogy (**Table S4**). ASTRAL species trees were estimated from the filtered gene trees using all 602 gene trees and using filtered sets of gene trees with at least 100 or 150 taxa, respectively. Local posterior probabilities were recorded as measures of support for each branch, and the polytomy test in ASTRAL III (Sayyari and Mirarab, 2018) was also applied.

For a supermatrix analysis, CSC orthogroup alignments were concatenated into DNA and protein supermatrices using FASconCAT (Kuck and Meusemann, 2010). Phylogenetic trees were estimated from the concatenated alignment including all 602 single copy gene alignments using RAxML v. 8.2 (Stamatakis, 2014). DNA alignments were partitioned by codon position, where the first and second codon positions were made into one partition, and the third codon position was a second partition. In addition, concatenated trees were also run with gene-based partitioning, where each gene was treated as a separate partition. We used GTRGAMMA for modeling rate variation of the DNA sequences. In addition to super matrix analyses including all 602 CSC orthogroups, analyses were performed on subsets that retained the 100 and 150 orthogroups with greatest species representation.

We further explored the placement of Asparagales and Liliales, which conflicted with plastid-based studies (see below), using, for computational efficiency, a subsample of 67 species and 1,375 BUSCO genes (Simão et al., 2015). For each taxon, only BUSCO sequences that had a single transcript were used for phylogenomic analysis, leaving missing data in places where multiple sequences were recovered from a single sample. Multiple sequence alignment and tree estimation were performed as described above. Species trees and clade support were estimated from the gene trees using ASTRAL III (Zhang et al., 2018). In order to understand how the CSC genes compared with the BUSCO sets, enrichment clustering was run with DAVID (Huang et al., 2009) for *Arabidopsis* sequences sorted to BUSCO sets and CSC sets separately. The BUSCO sets were also separated into those classified into the same or different orthogroups as the monocot conserved CSC genes.

### Concordance analysis

To further explore patterns of support and conflict for coalescent-based relationships, we calculated both gene and site “concordance factors” in IQ-TREE v. 2.2.0 (gCF and sCF, respectively; Baum, 2007; Minh et al., 2020a; Minh et al., 2020b). Branches may receive 100% bootstrap support or posterior probabilities of 1.0, yet these measures of sampling variance (Felsenstein, 1985) may obscure patterns and potential processes contributing to genealogical discordance. The gCF summarizes the proportion of ‘decisive’ individual gene trees containing a particular branch in the specified reference tree (here, the species tree inferred by ASTRAL). The sCF summarizes the average proportion of sites decisive for a particular branch in the reference tree concordant for that branch, averaged across 1000 subsampled quartets (Minh et al, 2020b). Here, ‘decisive’ denotes that a site is parsimony-informative for a particular quartet, yet decisive sites can be either concordant or discordant with a particular branch, and thus sCF represents the proportion of concordant sites relative to decisive sites. IQ-TREE 2.2.0 takes as input the reference (i.e., ASTRAL) species tree estimate, all gene trees, and all gene alignments, and produces a table with gCF, sCF, and other information for each branch, including ‘discordance factors.’ Discordance factors gDF_1_ and gDF_2_ summarize the proportion of genes concordant with the nearest-neighbor relationships of a particular branch in the reference tree, while gDF_P_ (‘paraphyly’) summarizes all other discordance. Further, we tested the expected pattern under a scenario of incomplete lineage sorting (ILS) using a chi-square test, with the null hypothesis being that the number of genes or sites supporting the two nearest-neighbor relationships for a node should be roughly equal (represented by P-values for gEF and sEF. gCF and sCF were plotted along with LPP for each branch of the ASTRAL species tree estimate, using ggplot2 v.3.3.5 (Wickham, 2016).

## Results

### Transcriptomes assembly and single copy assignment

We started with a set of 4.1 billion paired-end transcript fragment reads averaging 24 million pairs of raw reads per sample. Following adapter removal and quality trimming, an average of 21.1 million pairs of reads were recovered per transcriptome and used for *de novo* assembly (**Table S1**). The *de novo* assembly files contained an average of 86,211 contigs + singletons (median = 75,241). These sequences (scaffolded contigs + singletons) had a mean length of 745 bases and N-50 length of 1,161 bases (medians = 715 and 1,098. Bases, respectively). An average of 60,679 (median = 58,712) coding DNA sequences and inferred protein sequences were recovered per transcriptome following translation by ESTScan. This number dropped to 57,126 contig sequences (median = 55,582) after post processing and deduplication using genome tools (Gremme et al., 2013). The mean and median N-50 lengths for these deduplicated sequences was 935 and 959 bases, respectively.

**Figure 1:**
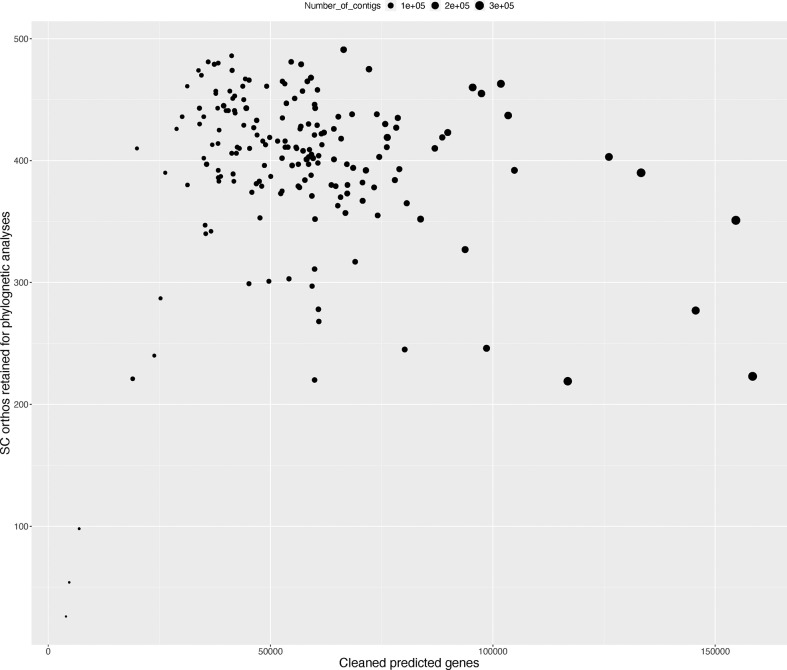
Summary of CSC orthogroup representation in transcriptomes used in the phylogenetic analysis. Figure shows the number of CSC genes retained for phylogenetic inference (Y-axis) versus the total number of translated and deduplicated genes after filtering (X-axis). Dot size corresponds to the total number of Trinity assembled contigs for each sample.

On average, 537 (median – 560) of 602 CSC orthologs were recovered per transcriptome, but after scaffolding, removing taxon-specific duplicated genes, unscaffolded alternative splice variants of unduplicated genes, and short transcripts using the PlantTribes toolkit (https://github.com/dePamphilis/PlantTribes), an average of 395 single copy genes per transcriptome (median = 410) were retained. Only 17 transcriptomes retained 301 or fewer single copy genes after the post-processing steps (**Figure 1** and **Table S1**) with *Helmholtzia* retaining the fewest, with just 26 CSC gene assemblies.

### Phylogenetic inferences

**Figure 2:**
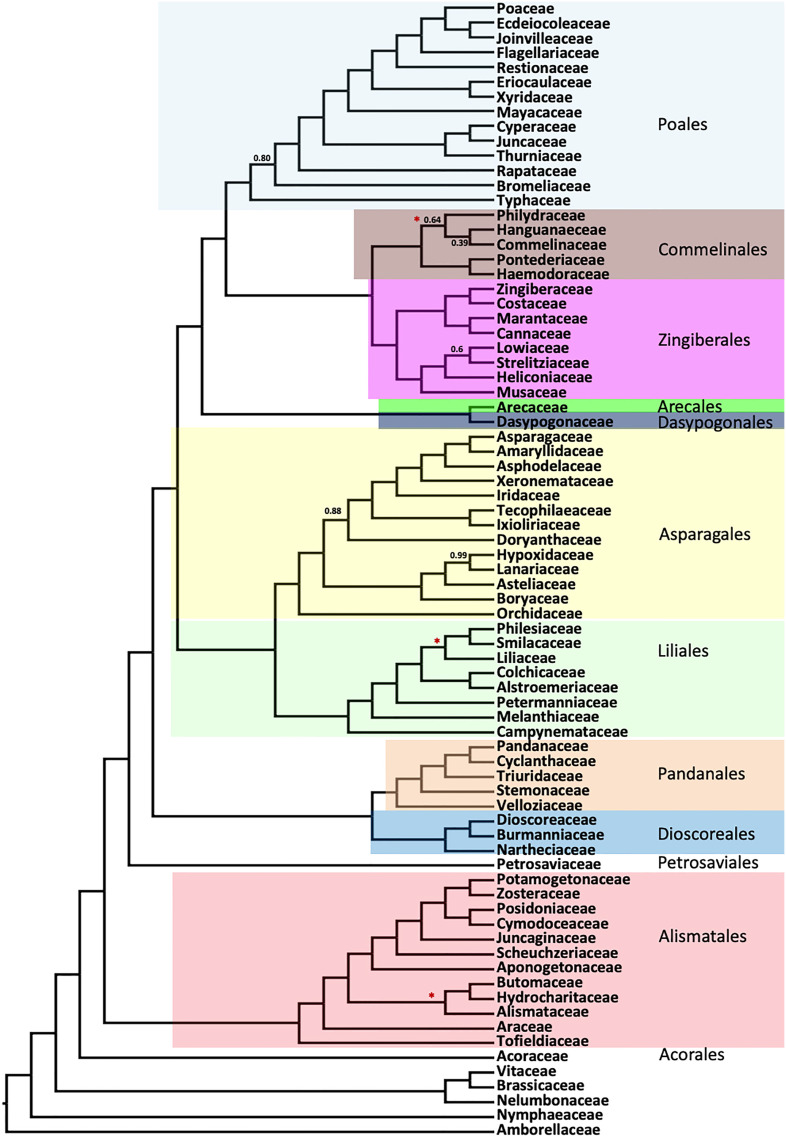
Summary of the order- and family-level clades inferred using ASTRAL coalescent analyses with 602 single copy genes. Numbers indicate the local posterior probability (LPP) for the main topology, for any LPP value less than 1.0. Tree topologies from RAxML concatenated analyses (**Figure S1**) are identical to this summary, except where a red star (“*”) indicates a difference between the two trees (specific differences are shown in detailed **Figures 3**–**5**).

The ASTRAL species trees and RAxML supermatrix trees were nearly identical as summarized in **Figures 2** and **S1**. Both analyses yielded strong support across most of the tree. Topologies were identical at the ordinal level and nearly identical within familial levels when different stringencies of filtering (based on completeness), and or different data partitioning schemes were used, and so we focused on the presentation of results on the full nucleotide alignments and with partitions based on codon positions.

**Figure 3:**
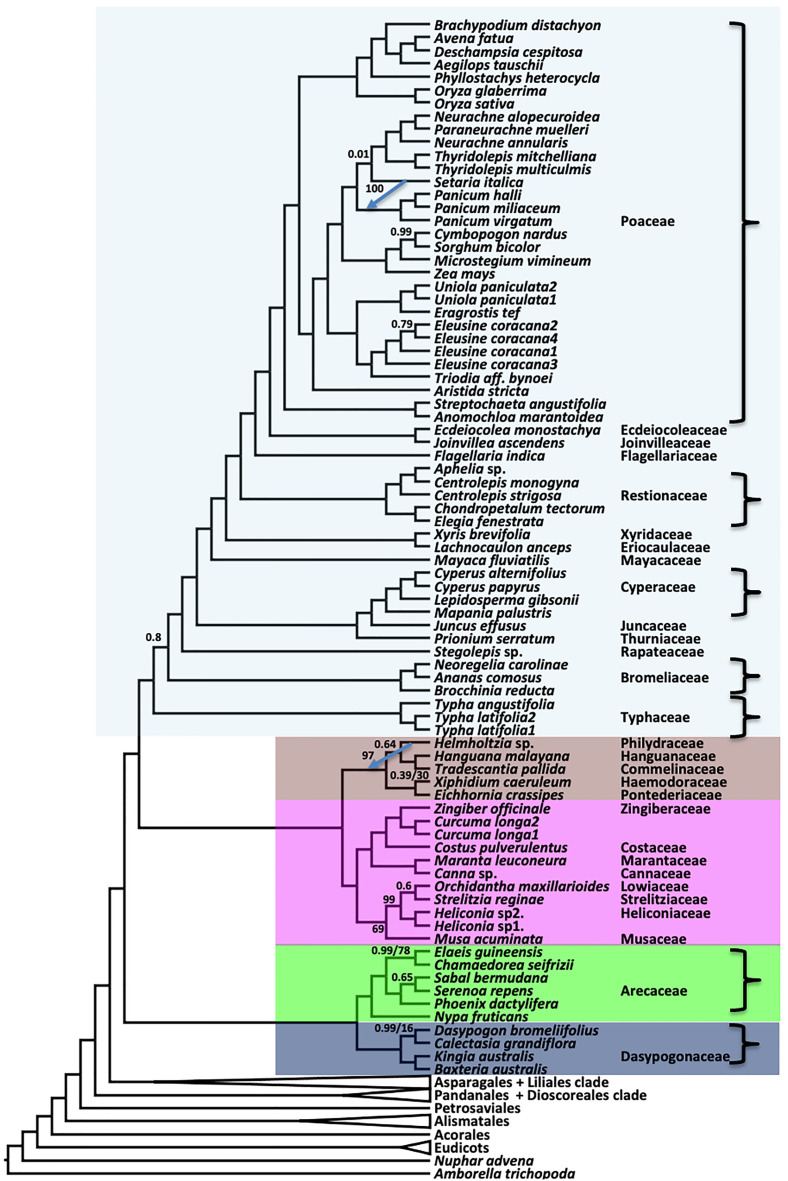
Relationships in the commelinid clade inferred using ASTRAL coalescent analyses with 602 single copy genes. RAxML topology is identical, except for the branches indicated with a blue arrow, where the tip of the arrow shows the position of the indicated branch in RAxML. Ordinal color scheme same as **Figure 2**. All support values have an LPP=1.0 and BS=100%, unless indicated in the diagram.

Inter-ordinal relationships within the commelinid clade are identical between the coalescent (ASTRAL) and concatenated (RAxML) analyses, with posterior probability (LPP)) of 1.0 for the former and 100% bootstrap support (BS) for the latter (**Figures 3**, **S1**). Within Poales, the position of *Setaria* differs between the coalescent (**Figure 3**) and concatenation trees (**Figure S1**), though with weak support (LPP 0.01) in the former and a strong support (BS 100%) in the latter. Typhaceae are resolved as sister to a clade comprising the remainder of the order Poales with strong support. A clade comprising Commelinales and Zingiberales is sister to Poales in both the ASTRAL and supermatrix RAxML trees. Arecales and Dasypogonales comprise a clade that is sister to the rest of the commelinids. The relationships within Dasypogonales and Arecales were identical between the RAxML and ASTRAL trees.

**Figure 4:**
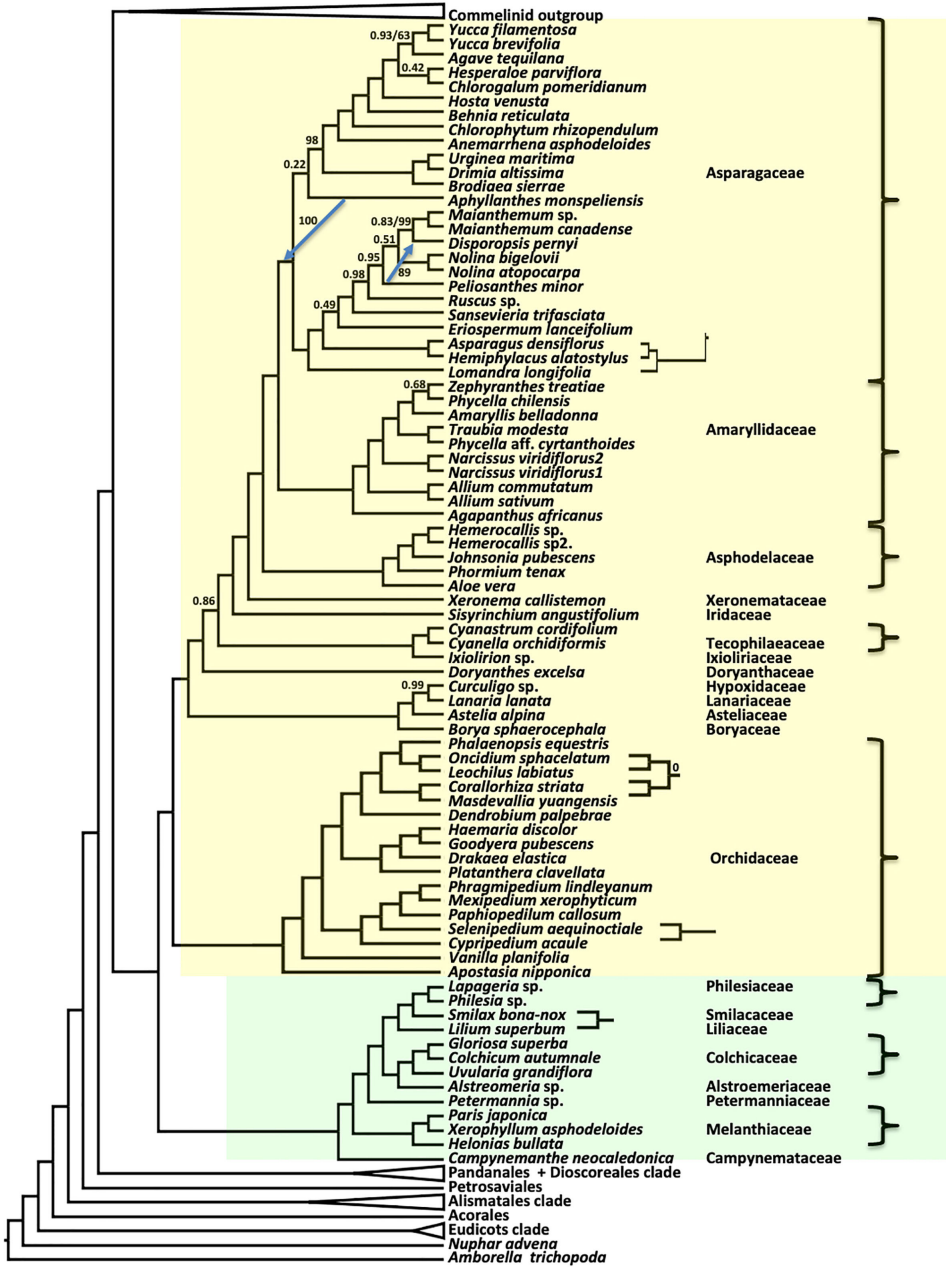
Relationships in the Asparagales (yellow) + Liliales (green) clades inferred using ASTRAL coalescent analyses with 602 CSC genes. Ordinal color scheme same as **Figure 2**. RAxML topology is identical, except for four small subtrees shown on the right, and two blue arrows showing different branch positions in the RAxML tree. All support values have an LPP=1.0 and BS=100%, unless indicated in the diagram.

As seen in previous species tree estimates using nuclear genes (Zeng et al., 2014; McKain et al., 2016; OTPT Initiative, 2019; Baker et al., 2022), Asparagales and Liliales formed a clade in both coalescent (ASTRAL) and concatenated supermatrix (RAxML) trees (**Figure 4**). In the ASTRAL tree, seven nodes in the Asparagales + Liliales clade had local posterior support values less than 0.9, while all but five nodes were fully supported in the concatenated analysis (**Figure 4**). There were a few topological differences between the two analyses, often at nodes that received less than full support in one of the trees: (1) *Lomandra* was placed as the sister of the Asparagoideae clade (the latter including *Asparagus* and *Hemiphylacus)* in the concatenated analysis, whereas it is sister to a larger clade in the coalescent analysis; (2) Within Asparagaceae, the positions of *Peliosanthes minor* and *Aphyllanthes monspeliensis* differ; (3) *Cypripedium* and *Selenipedium* formed a clade in the concatenated analysis, but *Cypripedium* was sister to other slipper orchids (*Phragmipedium*, *Mexipedium*, *Paphiopedilum*, and *Cypripedium*) in the ASTRAL tree; (4) The relationship among the four other orchids *Oncidium*, *Lechochilus*, *Corallorhiza*, and *Masdevallia* is also slightly different, although that relationship has BS of 0% in the concatenated analysis and LPP of 1 in the coalescent tree (5) *Smilax* and *Lilium* were sister taxa in the concatenated analysis, but *Smilax* was sister to a clade comprising *Philesia* and *Lapageria* in the ASTRAL analysis. Both the ASTRAL and concatenated analyses resolved Doryanthaceae as sister to a clade including Ixioliriaceae-Tecophilaeaceae, Iridaceae, Xeronemataceae, Asphodelaceae, Amaryllidaceae and Asparagaceae, with the latter seven-family clade well supported in the ASTRAL analysis (LPP 0.86) as well as in the concatenated analysis (**Figure 4**). Campynemataceae were resolved as the sister to the remainder of the Liliales with strong support in both analyses. The recently published analysis of Baker et al. (2022) using the Angiosperm353 bait set (Johnson et al., 2019) resolved Petermanniaceae, Campynemataceae and Melanthiaceae, as successive sister clades to the remainder of the Liliales, but the LPP for the Campynemataceae + remaining Liliales clade was quite low (0.59) there. Our study (**Figures 2**, **4**, **S1**) and that of Baker et al. (2022) provide maximum support for the placement of Melanthiaceae as sister to a clade comprising all Liliales families other than Campynemataceae, whereas the plastome analysis (Givnish et al., 2018) placed Melanthiaceae sister to the following clade: (Smilacaceae, (Liliaceae, (Philesiaceae, Ripogonaceae))).

**Figure 5:**
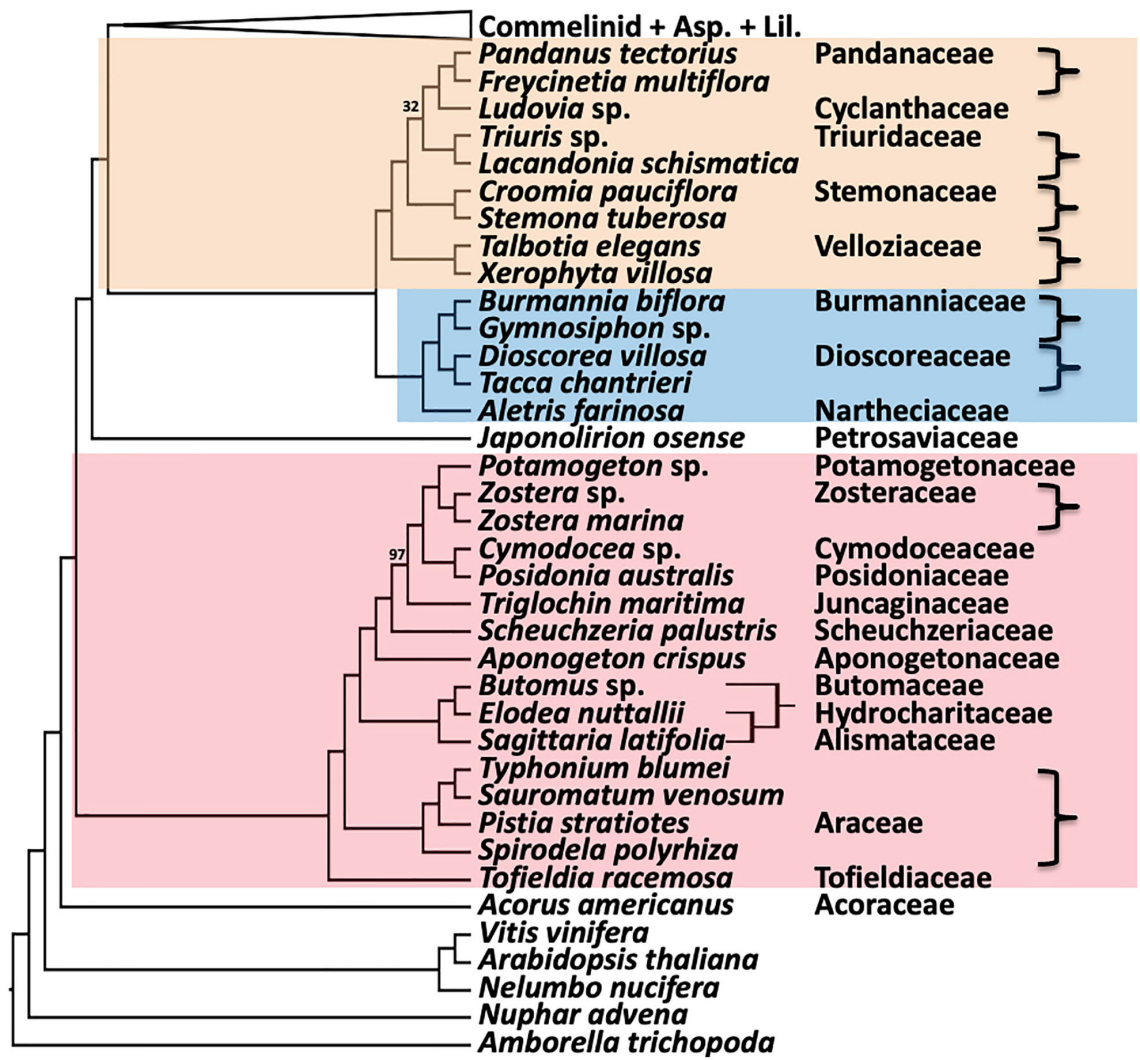
Relationships in the remaining five orders inferred using ASTRAL coalescent analyses with 602 CSC genes. Ordinal color scheme same as **Figure 2**. RAxML topology is identical, except for resolution of relationships among *Butomus*, *Elodea*, and *Sagittaria* (RAxML subtree shown on the right). All support values have an LPP=1.0 and BS=100%, unless indicated in the diagram.

The remaining inter-ordinal and inter-familial relationships were strongly supported (all LPP of = 1.0 and all but two BS of 100%; **Figure 5**). The order Pandanales had identical topologies between the ASTRAL (**Figure 5**) and RAxML trees (**Figure S1**), and only one weakly supported branch in the RAxML tree (BS of 32%) regarding the placement of Triuridaceae. Similarly, there was no difference between the two trees for Dioscoreales and the placement of Petrosaviales. All analyses placed Tofieldiaceae as sister to a clade comprising all other Alismatales taxa, as seen in the phylogenomic analyses of Ross et al. (2016); Baker et al. (2022) and Chen et al. (2022). Also in agreement with both plastome and nuclear gene phylogenomic analyses, the order Acorales was sister to all other monocot orders.

**Figure 6:**
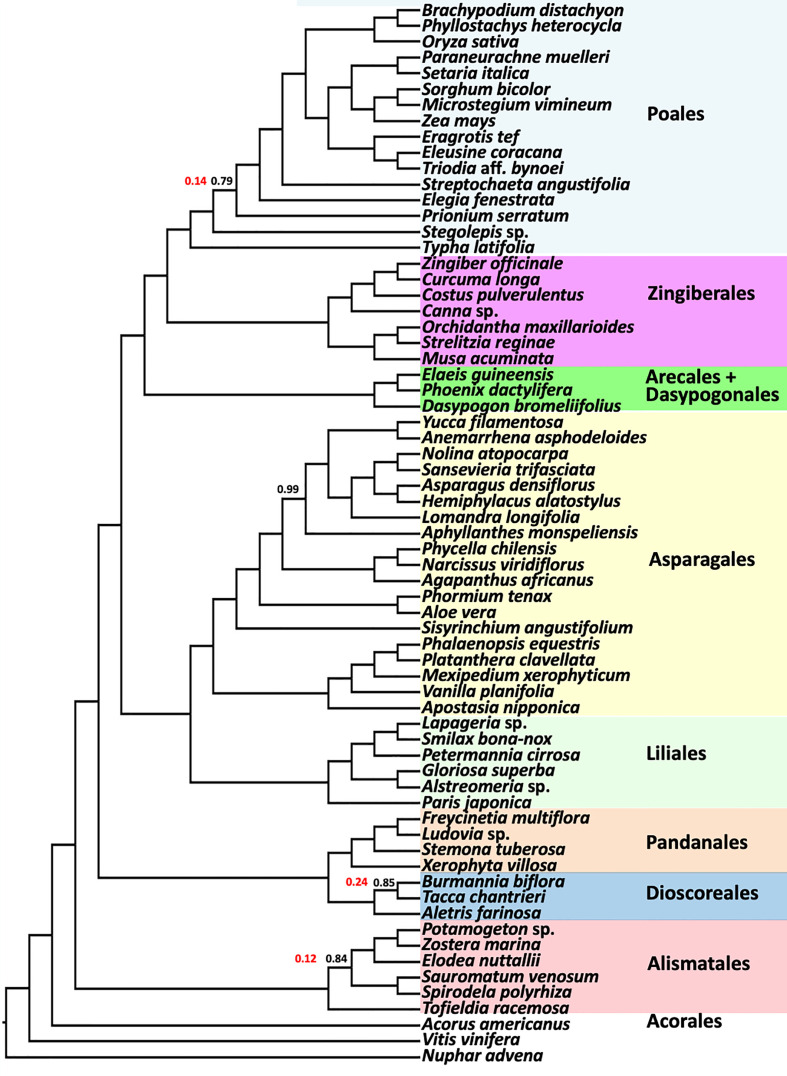
ASTRAL coalescent analysis of a subsample of 67 taxa using 1375 BUSCO genes. Ordinal color scheme same as Figure 2, showing the same relationships. All values have a LPP=1.0, except as indicated in black. Polytomy test result p-values are shown in red for branches where the null hypothesis of a polytomy is not rejected. Polytomy is rejected (p-value of 0) for all other nodes.

The BUSCO-based coalescent tree based on 1375 nuclear universal single copy orthologs was consistent with the results from the larger analyses. Analysis of the BUSCO genes resolved the Asparagales+Liliales clade (**Figure 6**), and all the ordinal relationships were also identical to results from the 602 single copy gene analyses. All branches except four had local posterior probabilities of 1.0 and only one branch had weak support (LPP=0.79). The polytomy test (Sayyari and Mirarab, 2018) rejected the null hypothesis of polytomy for all but 3 nodes. Also, the positions of all families in all orders were identical to the CSC analyses (**Figures 2**-**4**) except within Poales and Asparagales.

### Comparison between the CSC and BUSCO gene sets

Functional annotation clustering of CSC and BUSCO gene sets showed similarly enriched clusters between the two datasets (**Tables S5**, **S6**). The most enriched clusters contained the same uniprot keywords: Transit peptide, Chloroplast, plastid, DNA repair, DNA damage, methyl transferase, Helicase, DNA replication, DNA-binding, TPR repeat etc., indicating the photosynthetic, plastidic, and household nature of these gene sets. CSC and BUSCO gene sets had no significant differences in their enrichment patterns, meaning they were functionally indistinguishable. However, the specific genes present in the two gene sets were quite different. Overlap analysis between CSC and BUSCO showed that, out of the 1373 BUSCOs present in the Arabidopsis genome, 291 belonged to the CSC gene set (21.2% of 1373), and were single copy in all the scaffold genomes, while 1082 (78.8% of 1373) were not single copy genes in monocots (**Tables S7**, **S8**). The BUSCO gene sets that were not single copy had an overall average of 1.24 copies per genome, with gene numbers ranging from 0.58 to 28.9, implicating lineage-specific loss and retention of BUSCO genes following duplications. In an extreme case, as many as 59 genes were annotated in the genome of a single taxon (*Aegilops tauschii)*. Our analyses revealed that only a small fraction of BUSCO genes are actually single copy in this broad sampling of monocot genomes, while others were highly duplicated gene families. 313 genes that were exactly single copy in each of the 12 scaffold genomes (and therefore included in the CSC set) were not present in the BUSCO gene set (**Table S9**).

### Measurement of gene and species trees concordance and discordance

**Figure 7:**
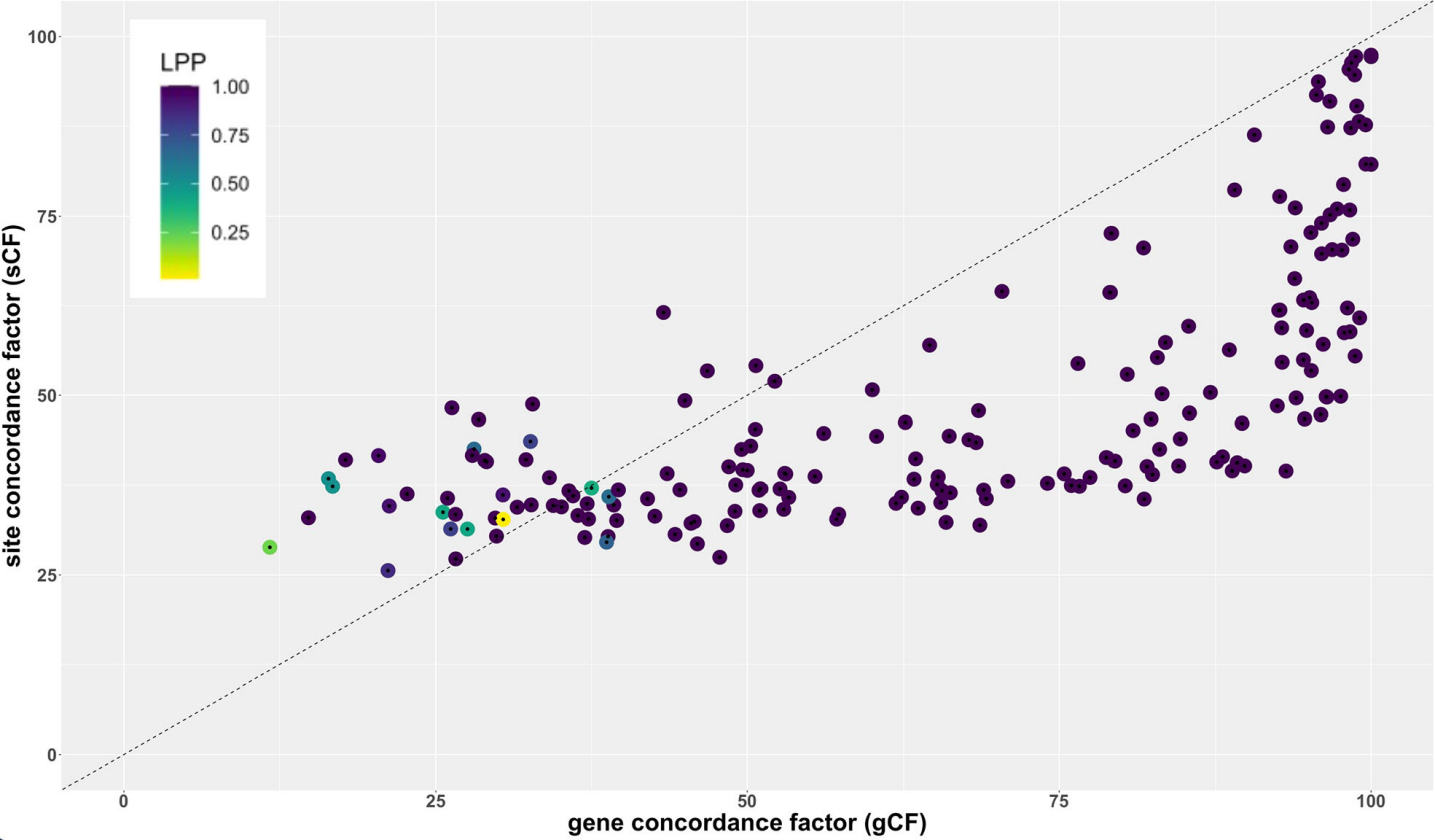
Relationship between site concordance factor (sCF), gene concordance factor (gCF) and LPP in the ASTRAL-based coalescent tree used in the analyses (**Figures 2**–**5**).

**Figure 7** shows the relationship between gene concordance factor (gCF), Site concordance factor (sCF), and branch support (LPP, local posterior probability) for all internal branches of the tree inferred with ASTRAL. All branches above a gCF of ~30 and sCF ~50 had LPP of 1.0 (**Table S10**). However, some branches with LPP = 1.0 had low gCF and sCF values, with the lowest gCF value for a branch with LPP = 1.0 of ~15 (node 293 **Table S10**, **Figure S3**) and the lowest sCF values for a branch with LPP = 1.0 at ~27 (nodes 243, 280). Overall, 66.3% of branches had a gCF value >50, meaning more than half of all genes are concordant for that particular branch (**Figure 7**; **Table S10**). 87.7% of branches had a sCF value > 33 indicating that there is a predominant signal across sites for most branches. Internal branch lengths from the tree inferred by ASTRAL are strongly correlated with gCF and sCF (Pearson’s r = 0.88, p < 0.0001; r = 0.77, p < 0.0001, respectively). Similarly, internode certainty was strongly correlated to both gene concordance factors and branch lengths (**Figures S5-S6**).

The branch indicating a sister relationship among Liliales and Asparagales had LPP = 1.0 but low values for gCF (22.7) and sCF (36.3). Gene discordance factors for the two nearest-neighbor relationships for this branch were also low (gDF_1_ = 8.2, gDF_2_ = 12.2), whereas the gDF_P_ had a value of 56.9, indicating that over half of all gene trees decisive for this branch were discordant with the ASTRAL species tree estimate and both nearest neighbor relationships (**Table S10** and **Figures S3-S4**). Site discordance factors for the two nearest neighbors at this branch were similar to the sCF for this branch in the ASTRAL tree, with 31.8 and 31.9% of all decisive sites being discordant. A chi-square test, however, failed to reject the null hypothesis of the pattern expected under incomplete lineage sorting (ILS) for genes and sites (gEF P-value = 0.029, sEF P-value = 0.98), underscoring the impact of rapid diversification and ILS at this branch (**Table S10** and **Figure S3-S4**).

The branch leading to the ‘commelinid’ clade had a relatively high concordance among genes, but relatively even concordance among sites for different topologies, although the null hypothesis under ILS was rejected considering sites concordant with the two nearest-neighbor relationships (gCF = 61.7, gDF_1_ = 0.2, gDF_2_ = 0, gDFP = 37.94; gEF P-value = 0.3; sCF = 35, sDF_1_ = 34.92; sDF_2_ = 29.9; sEF P-value < 0.001). Within the commelinid clade, the branch leading to ((Zingiberales, Commelinales), Poales) received relatively low gene and site concordance factors, and the null hypothesis expected under ILS was rejected for both genes and sites (gCF = 26.6, gDF_1_ = 18.9, gDF_2_ = 5.8, gDF_P_ = 48.7; gEF P-value < 0.0001; sCF = 33.4, sDF_1_ = 36.2; sDF_2_ = 30.3; sEF *P*-value < 0.001).

## Discussion

Our transcriptome-based analyses resolve and robustly support both ordinal and family-level relationships across monocot phylogeny. Aside from the strongly-supported resolution of an Asparagales+Liliales clade seen here and in other phylogenomic analyses of nuclear loci (Zeng et al., 2014; McKain et al., 2016; OTPT Initiative, 2019; Baker et al., 2022), our results support large-scale molecular analyses of monocot relationships based on plastome analyses. Notably our results corroborate inferences of Givnish et al. (Givnish et al., 2010; Givnish et al., 2018) and Barrett et al. (Barrett et al., 2013; Barrett et al., 2016b) with respect to long-standing questions regarding relationships among commelinid monocot orders. Poales is sister to Commelinales+Zingiberales in the so-called herbaceous clade, and Arecales (Arecaceae) are sister to Dasypogonales (Dasypogonaceae) in the so-called woody clade (Givnish et al., 2018). However, while the Givnish et al. (2018) plastome analysis provided 74% bootstrap support for the sister relationship of Arecaceae and Dasypogonaceae, our evidence based on hundreds of nuclear loci strongly support that conclusion, with 1.0 LPP and 100% BS. Givnish et al. (2018) proposed that Dasypogonaceae should be recognized as order Dasypogonales (Givnish et al., 1999; Givnish et al., 2010), rather than being included in Arecales (as proposed by APG IV 2016), because the two families are highly distinctive, share few if any potential morphological synapomorphies other than a “woody” habit (making it very hard to diagnose an order containing both), and diverged earlier (119 Mya) than any other pair of sister families among the monocots.

Of the five families with placements in our nuclear phylogenies that differ from those in the plastome tree (Givnish et al., 2018), three are among those with the weakest levels of support for familial placement based on the plastome data: Tofieldiaceae (35% BS for supporting node in Givnish et al., 2018), Philydraceae (50.6% BS) and Typhaceae (62.6% BS). Each of these weakly supported nodes in the plastome phylogeny is resolved with 1.0 LPP in the current analysis, except for the placement of Philydraceae which has a LPP of 0.6 in ASTRAL tree. The poor resolution for the placement of Philydraceae are not surprising given that we only recovered 26 CSC genes in the small RNA seq dataset for *Helmholtzia*. As expected based on simulation-based experiments for phylogenomic studies (Molloy and Warnow, 2018), removing *Helmholtzia* had no impact on other inferred relationships here (**Figure S6**). Moreover, a recent comprehensive phylogenomic analysis of the Commelinales using the Angiosperm353 bait set (Zuntini et al., 2021) also placed Philydraceae sister to the Hanguanaceae+Commelinaceae clade with only slightly higher support in the multispecies coalescent analysis (LPP = 0.74) and good support in the concatenated analysis (96% BS).

Concordance analysis for the placement of Tofieldiaceae as sister to the remainder of Alismatales showed that most genes were concordant (62.3%), and only a few (2.9% + 5.6% (NNI1+NNI2) were discordant with the estimated topology. As mentioned above, other phylogenomic analyses of nuclear loci also recover strong support for Tofieldiaceae as sister to a clade including the remainder of Alismatales (Baker et al., 2022; Chen et al., 2022). Similarly, the placement of Typhaceae as sister to the remainder of Poales is supported with good gene concordance (77.5%, discordance 0.4% + 0.2%) and previous phylogenomic inference (McKain et al., 2016), although Baker et al. (2022) recovered a Typhaceae+Bromeliaceae clade using the Angiosperm353 bait set.

Interestingly, the placement of Musaceae (represented by *Musa acuminata)* as sister to a clade comprising Heliconiaceae, Lowiaceae and Strelitziaceae (LPP=1.0; BS=69%) is consistent with the plastome tree of Givnish et al. (2018) and phylogenomic analyses of nuclear genes (Carlsen et al., 2018; Baker et al., 2022; but see Sass et al., 2016), but this placement of the Musaceae had low gene concordance (28.4%) and high gene discordance (13% and 22%, for NN1 and NN2 placements, respectively) in the current analysis (**Table S10**). The concordance/discordance data together with the conflicting placement of Musaceae recovered by Sass et al. (2016) and earlier studies may be a consequence of reticulation in the early diversification of the Zingiberales. Relationships among the eight families of order Zingiberales have also been contentious, with studies recovering different relationships, even when employing large phylogenomic datasets based on plastomes or nuclear data (Kress et al., 2001; Kress and Specht, 2005; Barrett et al., 2014; Sass et al., 2016). Carlsen et al. (2018) did not rule out the possibility of a ‘hard polytomy’ at the base of Zingiberales, possibly representing a rapid, simultaneous radiation among the major lineages. Although a polytomy is rejected at the base of Zingiberales (**Figure 6**), quartet analysis finds no evidence to reject the null hypothesis expected under the coalescence model (ILS) for a scenario in which the major lineages of Zingiberales diverged nearly simultaneously (over a short time span).

Within Poales, we find 1.0 LPP and 100% BS and for Ecdeiocoleaceae as sister to Joinvilleaceae, in a clade that is sister to Poaceae (**Figure 4**). This resolution is consistent with the previous phylotranscriptomic analysis of McKain et al. (2016) and the Angiosperm353 bait capture analysis of Baker et al. (2022), but conflicts with the most complete plastome phylogeny to date (Givnish et al., 2018), which places Ecdeiocoleaceae as sister to Poaceae with 100% BS, and Joinvilleaceae sister to both with 98% BS. Concordance analysis shows that 85.3% of all gene trees support resolution of the Ecdeiocoleaceae as sister to Joinvilleaceae clade (**Table S10**, **Figure S3-S4**).

The commelinid clade is another interesting region of the monocot tree; plastomes provide moderate support for ([(Zingiberales, Commelinales), Poales], [Arecales, Dasypogonales]), and nuclear loci provide overall strong support for the same relationships (Givnish et al., 2010; Barrett et al., 2013; Givnish et al., 2018). However, our test failed to reject the null hypothesis expected under a simple coalescence process (ILS) for gene counts, but strongly rejected the null hypothesis for site counts (**Table S10**). This suggests that while individual genes seem to fit the expectation of ILS, sites across the genome do not, possibly reflecting differences in information content among the CSC gene loci. Taking a closer look at the commelinids, the ILS test strongly rejects the null hypothesis for the clades representing [(Zingiberales, Commelinales), Poales], for both genes and sites (**Table S10**), whereas these relationships are strongly supported by plastomes alone (e.g. Givnish et al., 2010; Barrett et al., 2013; Givnish et al., 2018). Rejection of the expected pattern of ILS for both genes and sites may suggest an alternative explanation for conflict among these orders, for example due to ancient reticulation, or the effect of whole genome duplication and differential loss of paralogous regions (e.g., the ‘sigma’ event in Poales vs. the ‘gamma’ event in Zingiberales; D’Hont et al., 2012; McKain et al., 2016; Li et al., 2021).

Liliales and Asparagales have been recovered as successive sister lineages to the commelinid clade in several analyses of plastid genes and genomes (Chase et al., 2000; Rudall et al., 2000; Chase et al., 2006; Graham et al., 2006; Chase and Reveal, 2009; Givnish et al., 2010; Soltis et al., 2011; Givnish et al., 2018). However, very few known morphological synapomorphies separate the two clades. Dahlgren et al. (1985), segregated the Liliales from other tepaloid monocots based on introrse anthers and tepal nectaries; Asparagales were distinguished from Liliales based on the phytomelan crust covering the seeds, which is absent in the Liliales, but is also absent from most Orchidaceae and certain succulent Asparagales (Bogler and Simpson, 1995; Zomlefer, 1999). Stevens (2017) points to only two frequently reversed traits potentially supporting a clade defined by Liliales, Asparagales, and the commelinid orders: cymose inflorescence branches and protandry. The single potential morphological synapomorphy for a clade formed by Asparagales and the commelinids is more dubious: long styles. Long style is a somewhat subjective character state, and orchids have highly modified, fused columns that are variable in length.

In fact, the lilioid group of monocots is complex and highly diverse, leading to confusion about exact placements (e.g., Cronquist and Takhtadzhian, 1981; Dahlgren et al., 1985; Chase et al., 1995). Both Asparagales and Liliales exhibit diverse growth forms, but similarities in reproductive or vegetative morphology among taxa in both orders have long been noted (Dahlgren et al., 1985; Goldblatt, 1995; Rudall et al., 2000). Dahlgren et al. (1985) considered the superorder Lilianae (including families in Dioscoreales, Asparagales, and Liliales) as monophyletic, but subsequent analyses using plastid genes and genomes rejected this. All analyses using nuclear genome-scale nucleotide and amino acid sequence alignments recover a strongly supported clade comprising Liliales+Asparagales. The plastid genome is inherited as a single linkage group comparable to a genetic locus (Doyle, 2022) and the apparent conflict between nuclear and plastome phylogenomic inferences could potentially be accounted for by rapid divergence and incomplete sorting of ancestral plastome variation. Discordance, presumably due to ILS, is also seen among the nuclear gene trees.

Overall comparison of gCF and sCF values indicate that most genes individually contain low information content (**Figure 7**), but together contribute to a highly resolved and supported coalescent ‘species tree.’ The sister relationship of Liliales and Asparagales is strongly supported but differs from relationships based on recent plastome studies, which place Liliales and Asparagales as successive sister lineages to the commelinids (Davis et al., 2004; Graham et al., 2006; Givnish et al., 2010; Barrett et al., 2013, Barrett et al.,2016b; Givnish et al., 2018). Analysis of sCF and gCF for the Asparagales+Liliales clade reveals a pattern that is in line with a coalescence process and ILS. Comparisons of quartet frequencies (**Figure S5**) are also consistent with expectations given a coalescence process with rapid diversification. The quartet frequency for the Asparagales+Liliales clade is 0.45 with similar frequency for the other two alternative resolutions, Asparagales+commelinids (0.29) and Liliales+commelinids (0.26). Therefore, the conflict between the nuclear gene-based species tree and the plastome tree (e.g. Givnish et al., 2018) is easily interpreted as random sampling of ancestral variation as the commelinid and Asparagales+Liliales lineages diverged. A recent mitochondrial genome based phylogenetic study focused on placing mycoheterotrophic lineages recovered Asparagales as sister to most monocots except Acorales and Alismatales (Lin et al., 2022); the authors speculated that this was due to sparse taxon sampling in this part of the tree.

As resolved in most previous molecular phylogenetic analyses, Dioscoreales and Pandanales form a clade and were sisters to the clade comprising commelinids and the Asparagales+Liliales clade. Relationships within Pandanales have also been controversial (Davis et al., 2004; Rudall & Bateman, 2006; Lam et al., 2015; Soto Gomez et al., 2020), perhaps due to increased substitution rates in the mycoheterotrophic Triuridaceae. The positions of Triuridaceae (Triuris, Lacandonia) and Stemonaceae (Croomia, Stemona) with respect to Pandanaceae (Pandanus, Freycinetia), Cyclanthaceae (Ludovia), and Velloziaceae (Talbotia and Xerophyta) were the same as has been seen in combined analyses of genes encoded in the plastid and mitochondrial genomes (Soto Gomez et al., 2020; **Figure 5**). Quartet frequencies estimated from gene trees in the ASTRAL analysis (**Figure S5**) are quite similar for the placement of the Pandanaceae+Cyclanthaceae clade sister to Triuridaceae (Q= 0.45) or Stemonaceae (Q=0.39), and the third alternative, Pandanaceae+Cyclanthaceae sister to a Stemonaceae+Triuridaceae clade has a significantly lower quartet frequency (Q=0.16). The skewed quartet frequencies are not expected given divergence under a coalescence model and may be due to biased gene flow after these three ancestral lineages diverged or possibly heterotachy associated with a shift from autotrophy to mycoheterotrophy in Triuridaceae. Both the ASTAL and RAxML tree estimates are also similar to published plastome trees (Givnish et al., 2018; Lam et al., 2018), which seem to have successfully placed several mycoheterophic taxa using plastome data despite multiple gene losses and relaxation of selection on plastid encoded photosynthetic genes (Lam et al., 2018). Mycoheterotrophic monocots have a nucleotide substitution rate for plastid genes that is 6.9 ± 4.1 times that of their green sisters, with *Thismia* plastid sites evolving 364 times faster than its close relative *Tacca* (Givnish et al., 2018). Clearly, the evolution of plastomes has been strongly affected by the shift to mycoheterotrophy, which could interfere with phylogenetic inferences, unless dense taxon sampling is available and large data sets are subjected to careful analysis (Lam et al., 2018). A recent study based on slowly evolving mitochondrial genomes (Lin et al., 2022) also found the relationships among the five Pandanales families found here for the ASTRAL analysis (**Figure 5**), i.e., (Velloziaceae, (Stemonaceae, (Triuridaceae, (Pandanaceae, Cyclanthaceae)))), but with improved support.

The placements of Petrosaviales, Alismatales and Acorales were consistent with previous phylogenomic analyses of both plastid (Lam et al., 2016; Ross et al., 2016; Givnish et al., 2018; Lam et al., 2018) and nuclear genes (Zeng et al., 2014; OTPT Initiative, 2019). This study includes deeper sampling of Alismatales than previous phylotranscriptomic analyses including Tofieldiaceae (*Tofieldia*). The plastome phylogenies reported in Ross et al. (2016); Givnish et al. (2018), and Lam et al. (2018) generally had poor to moderate support for either Araceae or Tofieldiaceae as sister to the rest of the order (e.g., maximum of 76% support for Tofieldiaceae sister in Ross et al., 2016). In Givnish et al. (2018), the position of Tofieldiaceae had the weakest support of any family in the plastid phylogeny with a bootstrap support of less than 50% for being sister to all alismatids except Araceae. The plastome analysis by Givnish et al. (2018) indicates that the branches involved are very short, and very deep: the inferred stem age of Araceae was 123.96 Mya, and 123.56 Mya for Tofieldiaceae and the clade formed by the remaining 11 Alismatales families. Nonetheless, our analyses return strong support for this placement of Tofieldiaceae as sister to the remaining Alismatales.

Finally, the vast majority of BUSCO genes are not strictly single copy in monocots, suggesting that these genes may return to single copy following duplication more slowly than the strictly single copy gene set, increasing the chance of orthology misspecification with BUSCO genes. Nevertheless, BUSCO trees in this study were largely congruent with those based on CSC genes, and both gene sets have indistinguishable functional biases, suggesting that both are samples of a larger gene set that can both provide similarly strong evidence for phylogenomic analyses. Key data handling steps for both data sets were the removal of genes from any taxon that had more than a single gene, or were identified as “rogue” taxa based on their unusually long branch lengths, suggesting that these steps alone may minimize orthology misspecification.

## Data availability statement

The data presented in the study are deposited in the NCBI’s SRA repository under BioProject accessions PRJNA313089, PRJNA752894, SRP009920, PRJNA412930, and PRJNA752837; SRA accession IDs for each sample are reported in **Table S1**.

## Author contributions

PT, CdP, JL-M, TG, DS, CA, JP, JD, WZ, SG, and CB, contributed to conception and design of the study. DS, JL-M, JP, CdP, JM, JR, MM, KH, AH, MV, JC, NI, and BF performed fieldwork, obtained samples, and processed samples for transcriptome analysis. SA, JL-M, PT, EW, and CdP organized the database. PT, CdP, JL-M, EW, and CB performed analyses and created graphics. PT and CdP wrote the first draft of the manuscript. JL-M, CB, CA, TG, and JD wrote sections of the manuscript. All authors contributed to the article and approved the submitted version.

## Acknowledgments

Data generation was performed under the Monocot Tree of Life project (MonAToL), DEB-0829868, at Cold Spring Harbor Laboratories, University of Georgia, and Penn State University. We thank Sarah Johnson, Riva Bruenn, Nina Hobbhahn, and Peter Linder for tissue samples, and Lisa DeGironimo, Chang Liu, Charlotte Quigley, and Paula Ralph for assisting with RNA isolations. We thank Norman Wickett for early discussions about this work. PT and EW and computer resources for this paper were supported in part by DEB-0829868 and IOS-1238057, and by the Huck Institutes of the Life Sciences and Department of Biology at Penn State University. We also thank three reviewers for their helpful comments.

## Conflict of interest

The authors declare that the research was conducted in the absence of any commercial or financial relationships that could be construed as a potential conflict of interest.

## Publisher’s note

All claims expressed in this article are solely those of the authors and do not necessarily represent those of their affiliated organizations, or those of the publisher, the editors and the reviewers. Any product that may be evaluated in this article, or claim that may be made by its manufacturer, is not guaranteed or endorsed by the publisher.
