# Supplementary material for: Phylogenomic resolution of order- and family-level monocot relationships using 602 single-copy nuclear genes and 1375 BUSCO genes
Source: Front Plant Sci. 2022 Nov 22;13:876779. doi: 10.3389/fpls.2022.876779 (PMC9723157; doi:10.3389/fpls.2022.876779)
Supplement: Supplementary file 1 [file DataSheet_1.docx]

List of supplementary figures:

Figure S1. Phylogram of the RAxML concatenation tree partitioned by codon positions. Filtered alignments were concatenated after running TreeShrink on a per-species mode. Order level color schemes are the same as in the main text.

Figure S2. Astral coalescent tree showing the branch numbers for the discordance analysis. See table 10 for the detailed analysis report. Order level color schemes are the same as in the main text.

Figure S3. Relationship of gene concordance factor (gcF) and site concordance factor (sCF) with branch length in the ASTRAL based coalescent tree used in the analysis.

Figure S4. Pairplots between different important parameters in the congruence analysis. Abbreviations:gCF = gene concordance factor, gDF1, gDF2: gene discordance factors for NNI-1 and NNI-2 branches , gDFP = gene discordance factor due to polyphyly, gN: Number of trees decisive for the branch, sCF = site concordance factor, gIC = Internode certainty for genes.

Figure S5. Cladogram of the ASTRAL coalescent tree estimated with nucleotide sequences showing quartet summary results in the nodes. Order level color schemes are the same as in the main text.

Figure S6. ASTRAL tree estimated after removing *Helmholtzia sp*. from all gene sets. Resolution of relationships among all other taxa are unchanged relative to the ASTRAL tree estimated with *Helmholtzia sp*. included (Figures 2-5, S7).


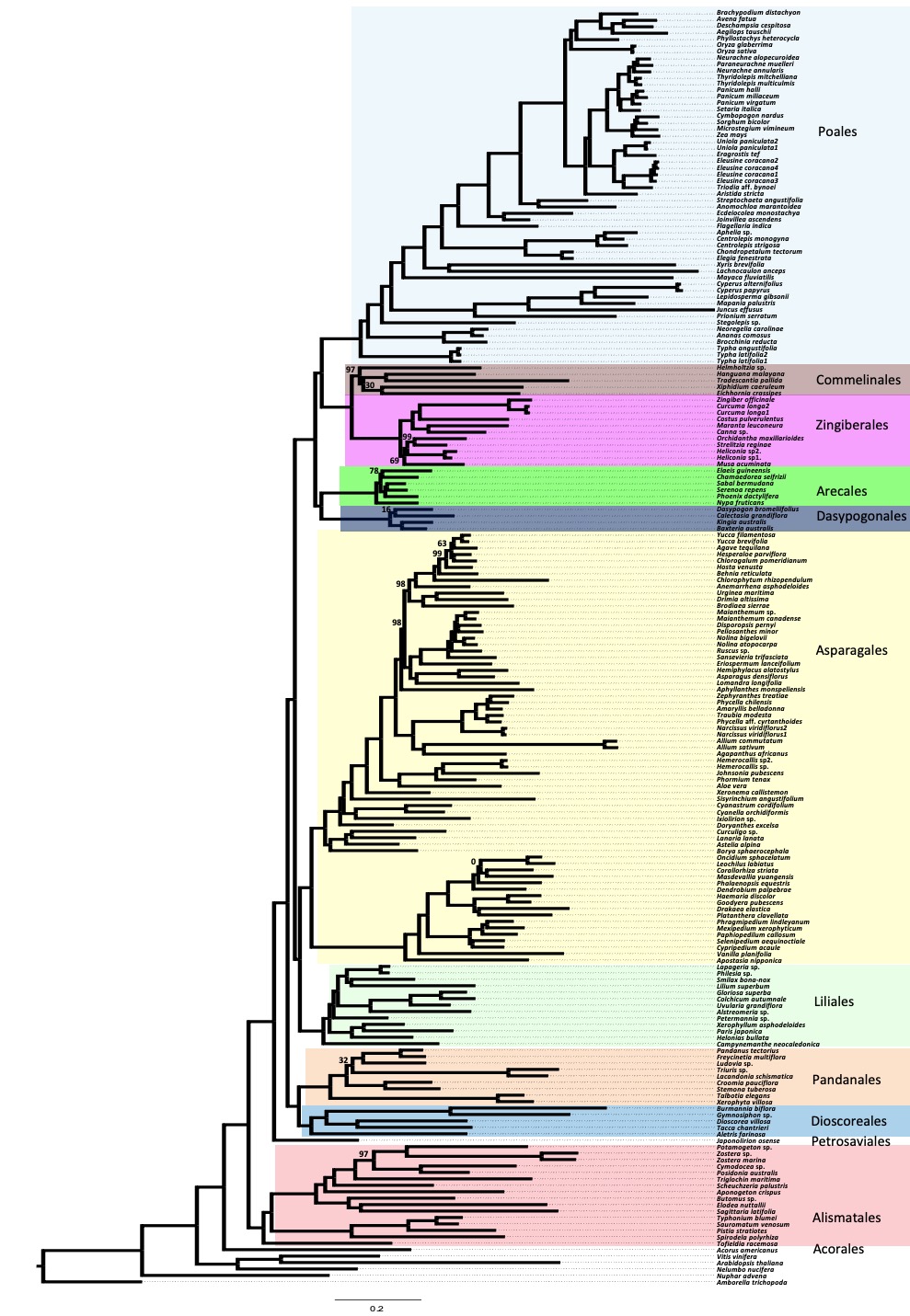


Figure S1. Phylogram of the RAxML concatenation tree partitioned by codon positions. Filtered alignments were concatenated after running TreeShrink on a per-species mode. Order level color schemes are the same as in the main text.


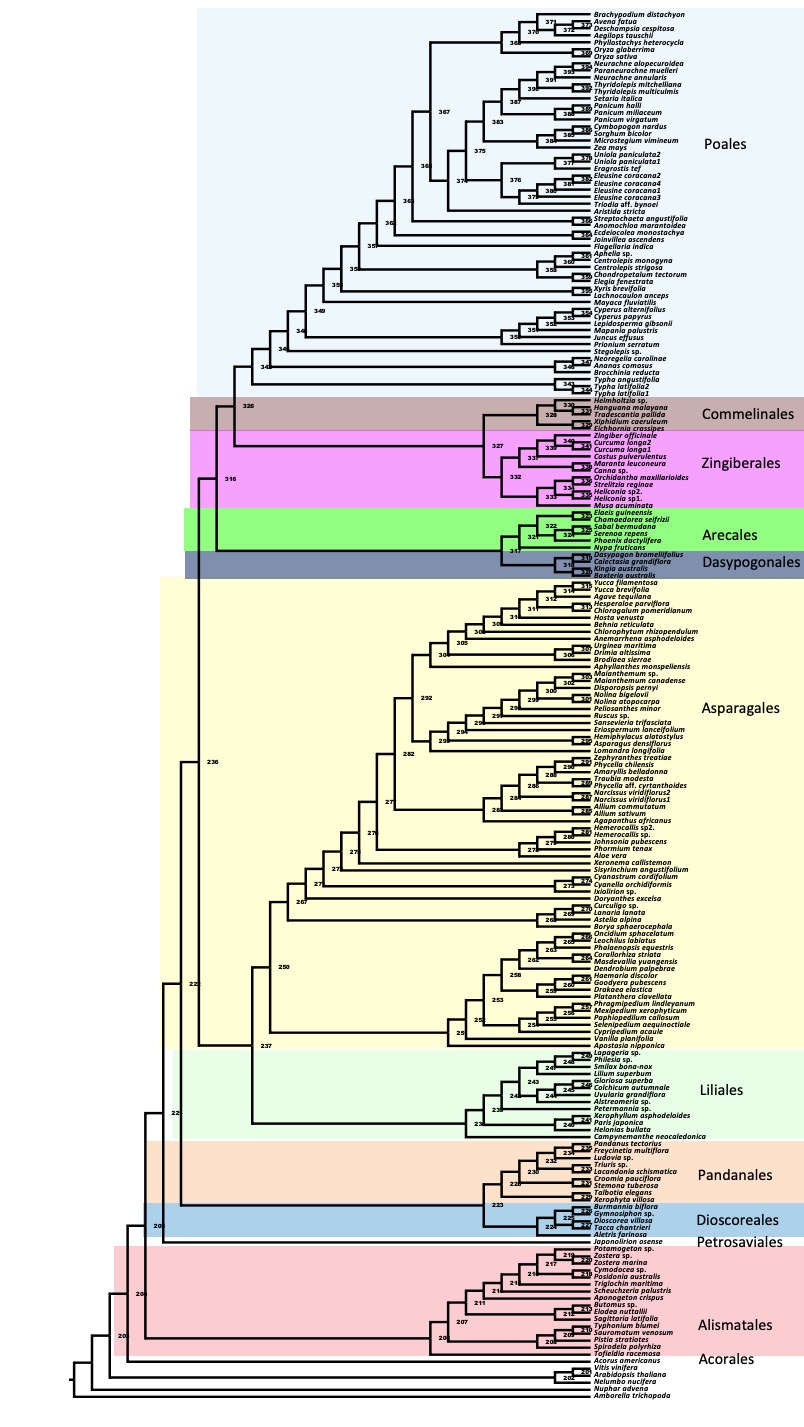


Figure S2. Astral coalescent tree showing the branch numbers for the discordance analysis. See table 10 for the detailed analysis report. Order level color schemes are the same as in the main text.


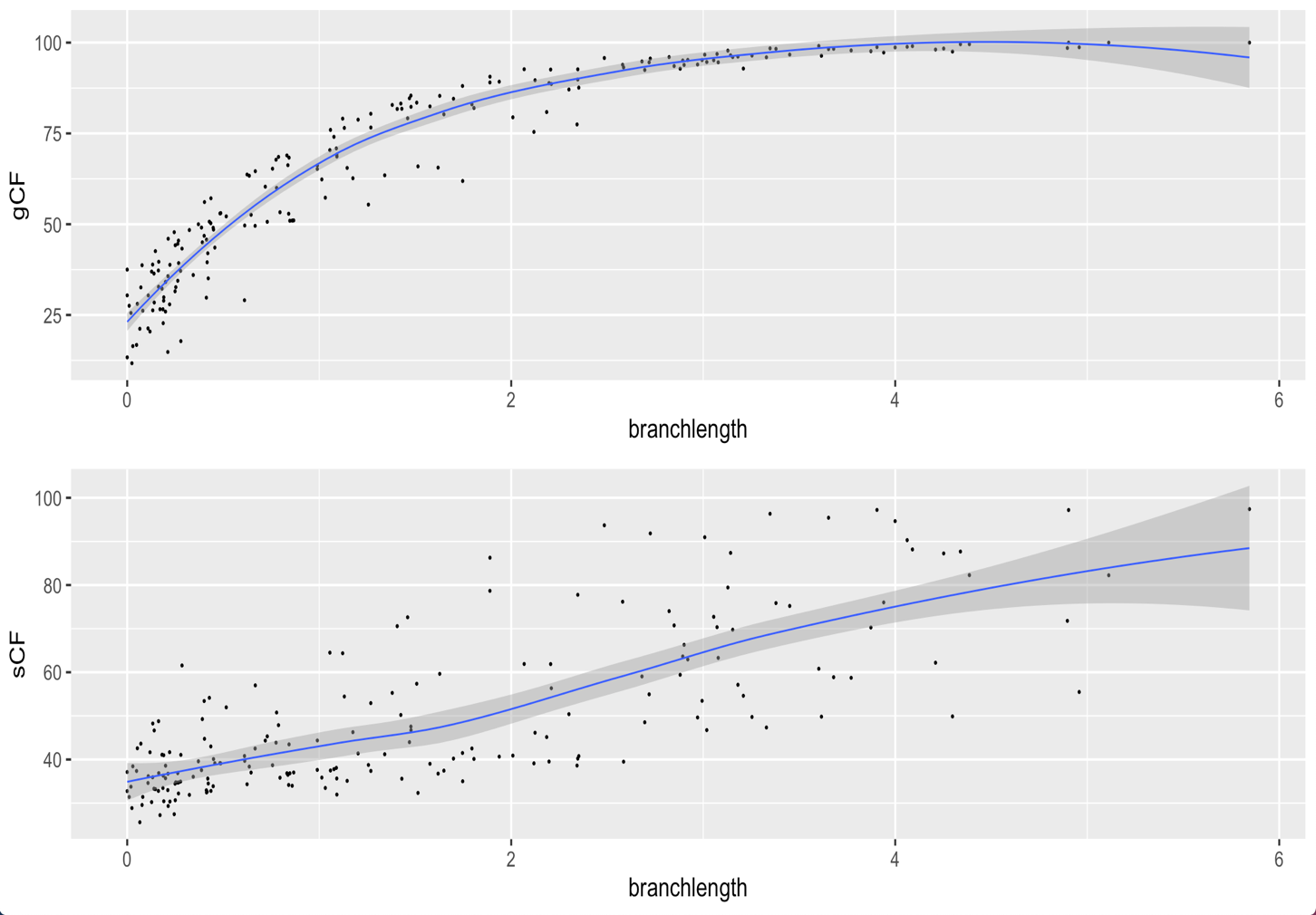


Figure S3. Relationship of gene concordance factor (gcF) and site concordance factor (sCF) with branch length in the ASTRAL based coalescent tree used in the analysis.


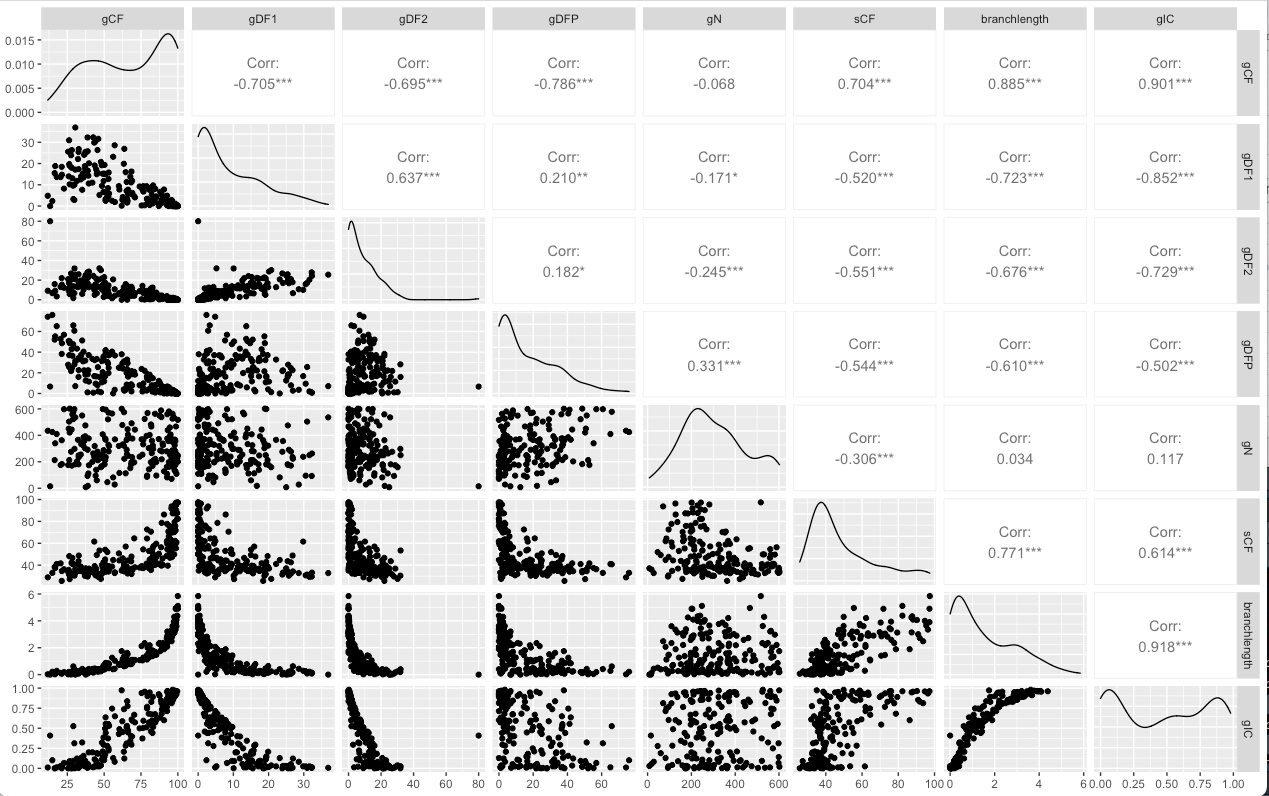


Figure S4. Pairplots between different important parameters in the congruence analysis. Abbreviations:gCF = gene concordance factor, gDF1, gDF2: gene discordance factors for NNI-1 and NNI-2 branches , gDFP = gene discordance factor due to polyphyly, gN: Number of trees decisive for the branch, sCF = site concordance factor, gIC = Internode certainty for genes.


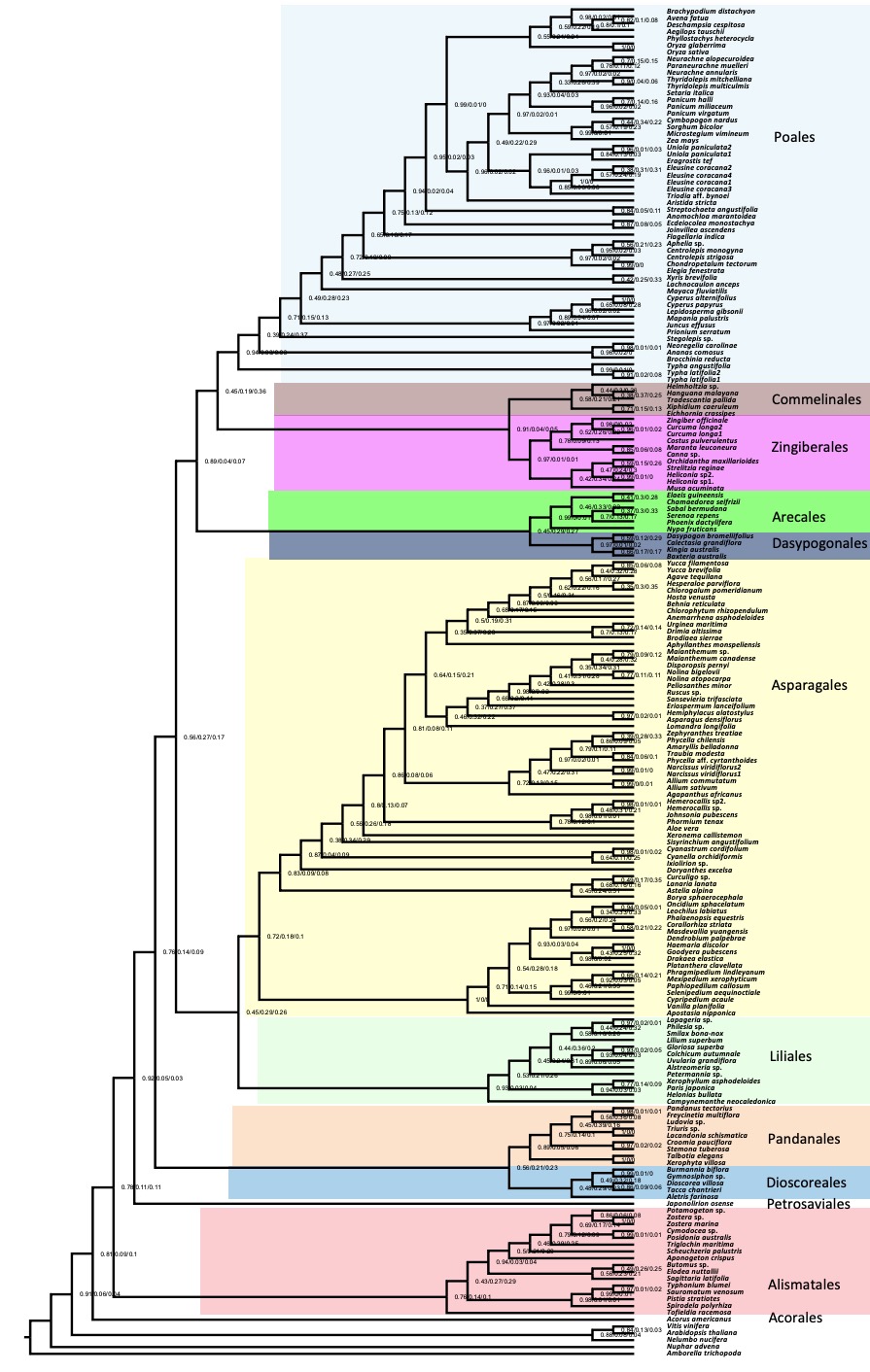


Figure S5. Cladogram of the ASTRAL coalescent tree estimated with nucleotide sequences showing quartet summary results in the nodes. Order level color schemes are the same as in the main text


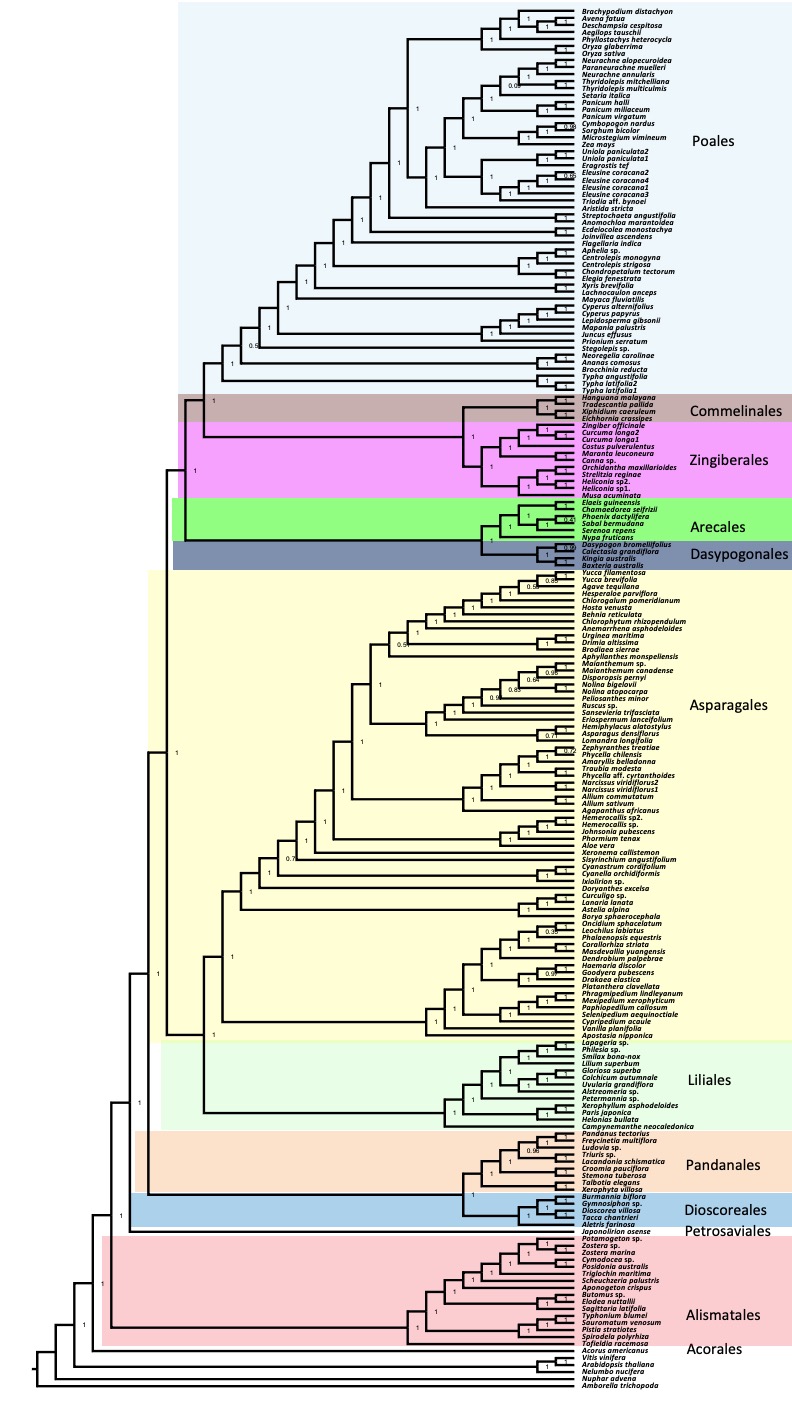


Figure S6. ASTRAL tree estimated after removing *Helmholtzia sp*. from all gene sets. Resolution of relationships among all other taxa are unchanged relative to the ASTRAL tree estimated with *Helmholtzia sp*. included (Figures 2-5, S7).
